# Cannabidiol in Developmental Epilepsy: Organoid-Guided Precision Medicine Across Critical Neurodevelopmental Windows

**DOI:** 10.3390/ijms27062899

**Published:** 2026-03-23

**Authors:** Jin Joo, Woo Sub Yang, Hyun Jung Koh

**Affiliations:** 1 Department of Anesthesiology and Pain Medicine, Seoul St. Mary’s Hospital, College of Medicine, The Catholic University of Korea, Seoul 06591, Republic of Korea; jiyo1004@catholic.ac.kr; 2 Department of Genetics, Yale Stem Cell Center, Yale School of Medicine, New Haven, CT 06520, USA; woosubyang@gmail.com

**Keywords:** cannabidiol (CBD), brain organoids, developmental epileptic encephalopathy, epilepsy, critical neurodevelopmental windows, precision medicine

## Abstract

Epilepsy is a progressive network disorder in which recurrent seizures drive maladaptive neurodevelopmental remodeling, cognitive decline, and pharmacoresistance, particularly in developmental epileptic encephalopathies. Cannabidiol (CBD) has emerged as an evidence-based adjunctive therapy for selected childhood-onset epilepsies; however, its broader clinical utility remains limited by heterogeneous responsiveness, restricted indications, and an incomplete understanding of developmental stage–specific efficacy and safety. Here, we synthesize molecular, preclinical and clinical evidence supporting the pleiotropic antiseizure and neuroprotective actions of CBD, including modulation of endocannabinoid-related G protein–coupled receptors, adenosine signaling, transient receptor potential channels, GABAergic maturation, and neuroinflammatory cascades. We highlight critical neurodevelopmental windows during which timely CBD intervention may exert disease-modifying effects by preventing pathological consolidation of hyperexcitable networks. Furthermore, we position human brain organoids as transformative translational platforms that recapitulate early human cortical development and epileptic network dynamics, enabling functional stratification of CBD-responsive phenotypes, developmental safety profiling, and precision therapeutic discovery within human-relevant neural circuits. Collectively, organoid-guided frameworks provide a mechanistic foundation for personalized, developmentally informed CBD therapy and advance precision medicine strategies aimed at modifying epileptogenic trajectories rather than solely suppressing seizures.

## 1. Introduction

Epilepsy is a chronic neurological disorder characterized by recurrent, unprovoked seizures requiring both rapid and sustained therapeutic control. Beyond transient clinical events, recurrent seizures act as cumulative neurobiological insults that drive persistent neuroinflammation, excitotoxic neuronal injury, maladaptive synaptic remodeling, and progressive network dysfunction. These pathological cascades contribute to cognitive impairment, behavioral disturbances, and reduced quality of life, particularly in patients with early-onset and drug-resistant epilepsy.

Despite the availability of numerous antiseizure medications (ASMs), approximately one-third of patients remain drug-resistant, underscoring the urgent need for novel disease-modifying therapeutic strategies [1,2]. In this context, cannabidiol (CBD), a non-psychoactive phytocannabinoid derived from *Cannabis sativa*, has emerged as a promising antiepileptic agent. Although its precise mechanism of action is not fully elucidated, growing evidence indicates that CBD exerts anticonvulsant and neuroprotective effects through multiple convergent molecular pathways, including modulation of GABAergic neurotransmission, activation of transient receptor potential vanilloid 1 (TRPV1) channel, inhibition of adenosine reuptake, regulation of intracellular calcium homeostasis, attenuation of neuroinflammatory signaling, and preservation of mitochondrial function.

A purified plant-derived CBD oral solution, commercially available as Epidiolex^®^ (USA)/Epidyolex^®^ (EU/UK) (GW Pharmaceuticals, a Jazz Pharmaceuticals company), was approved by the U.S. Food and Drug Administration (FDA) in 2018 for Dravet syndrome (DS) and Lennox–Gastaut syndrome (LGS), followed by approval for tuberous sclerosis complex (TSC)-associated epilepsy in 2021. In the Republic of Korea, Epidiolex^®^ was approved by the Ministry of Food and Drug Safety (MFDS) in 2019 and is currently distributed through the Korea Orphan & Essential Drug Center (KOEDC). Nevertheless, its real-world clinical accessibility remains limited due to restricted reimbursement policies, narrow regulatory indications, and responder rates that generally do not exceed approximately 50%, underscoring substantial unmet clinical needs [3].

Currently, CBD is used primarily as an adjunctive therapy for severe childhood-onset developmental and epileptic encephalopathies, particularly DS, LGS, and TSC, often in combination with clobazam [4,5,6]. While CBD is not intrinsically restricted to pediatric populations, regulatory approvals remain confined to specific syndromes supported by robust randomized clinical trials. Consequently, most epilepsy subtypes lack validated guidance regarding CBD responsiveness, optimal timing of intervention, and long-term developmental safety, limiting its broader clinical implementation.

Expanding the clinical indications of CBD and establishing reliable responder stratification strategies, developmental stage-specific efficacy profiles, and long-term safety data therefore represent critical unmet needs in epilepsy care. In this review, we summarize its advantages and limitations with conventional ASMs and discuss future directions for CBD-based epilepsy management, with particular emphasis on human brain organoid platforms as emerging translational and precision-medicine tools.

## 2. Literature Search Strategy

A comprehensive literature search was conducted using the databases: PubMed, Embase, Scopus, and Google Scholar, covering studies published from 2000 onwards. The following search terms were used: (“cannabidiol” OR “CBD”) *AND* (“epilepsy” OR “developmental epileptic encephalopathy” OR “drug-resistant epilepsy” OR “refractory epilepsy”) *AND* (“brain organoid” OR “cerebral organoid” OR “hippocampal organoid”) AND (“pregnancy” OR “maternal-fetal” *OR* “prenatal exposure”). Reference lists were also screened manually to identify studies relevant to CBD mechanisms, developmental stage-specific epileptogenesis, maternal-fetal neurodevelopmental safety, and organoid-based epilepsy modeling. This review was not conducted as a systematic review; instead, the references were selected based on mechanistic, developmental, and translational relevance.

## 3. CBD Targets and Mechanisms in the Brain

### 3.1. Molecular Targets

Cannabidiol (CBD) is a pleiotropic neuromodulator that exerts anticonvulsant and neuroprotective effects through interactions with multiple molecular targets rather than through classical cannabinoid receptor agonism. Unlike Δ9-tetrahydrocannabinol (THC), CBD displays minimal direct activity at CB1 and CB2 receptors and instead modulates a broad spectrum of non-canonical receptors, ion channels, transporters, and nuclear receptors involved in neuronal excitability and neuroinflammation.

**Figure 1:**
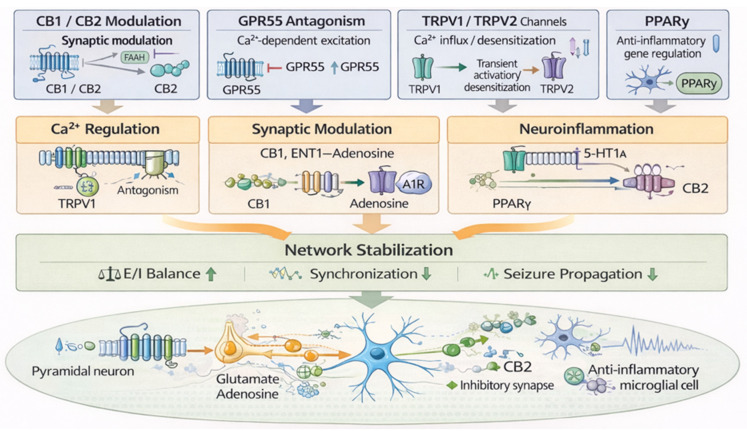
Molecular and Network Mechanisms of Cannabidiol (CBD) in Epilepsy. The molecular targets of CBD are functionally organized into three convergent axes: regulation of Ca^2+^-dependent excitability, synaptic modulation, and neuroinflammatory control. A1R, adenosine A1 receptor; ENT1, equilibrative nucleoside transporter-1; 5-HT, 5-hydroxytryptamine; GPR55, G protein-coupled receptor 55; TRPV, transient receptor potential vanilloid; PPARγ, peroxisome proliferator-activated receptorγ.

Rather than acting through a single dominant pathway, these diverse molecular interactions can be conceptually organized into three convergent functional axes: (1) regulation of Ca^2+^-dependent neuronal excitability, (2) modulation of synaptic transmission, and (3) control of neuroinflammatory and cellular stress responses. A schematic overview of these integrated mechanisms is presented in Figure 1. These axes are functionally integrated and collectively contribute to the stabilization of epileptic networks.

The principal molecular targets of CBD include G protein-coupled receptor 55 (GPR55), transient receptor potential vanilloid channels (TRPV1 and TRPV2 channels), equilibrative nucleoside transporter-1 (ENT1), serotonin 5-hydroxytryptamine (5-HT1A) receptors, peroxisome proliferator-activated receptor-γ (PPARγ), and multiple voltage-gated ion channels [7,8,9,10]. A structured overview of these molecular interactions, the principal targets of CBD and their functional implications in epileptic networks is summarized in Supplementary Table S1.

#### 3.1.1. Cannabinoid-Related Receptors

CBD-mediated modulation of the endocannabinoid system plays a central role in synaptic regulation and neuroinflammatory control.

CBD exhibits low orthosteric affinity for cannabinoid receptors CB1 and CB2 and does not act as a classical agonist. Instead, it functions as a negative allosteric modulator of CB1, altering receptor signaling without direct activation. In addition, CBD indirectly enhances endocannabinoid tone by inhibiting fatty acid amide hydrolase (FAAH), thereby increasing levels of the endocannabinoid anandamide. In the central nervous system, CB2 receptors are primarily expressed in microglia and immune-related cells. Modulation of CB2-associated signaling pathways contributes to the anti-inflammatory and immunoregulatory properties of CBD within epileptic brain tissue [7,10].

#### 3.1.2. GPR55 Antagonism

GPR55 signaling is closely linked to Ca^2+^-dependent excitatory transmission and represents a key component of CBD-mediated regulation of neuronal excitability.

GPR55 has emerged as a non-classical cannabinoid-responsive receptor implicated in neuronal excitability and synaptic transmission. CBD acts as a functionally relevant antagonist of GPR55.

Activation of GPR55 is associated with Ca^2+^-dependent glutamatergic signaling at excitatory synapses, and dysregulated GPR55 signaling has been observed in several experimental epilepsy models. Specifically, GPR55 expression is upregulated in excitatory neurons but downregulated in inhibitory interneurons. Antagonism of GPR55 by CBD may therefore contribute to the regulation of neuronal hyperexcitability [8,9].

#### 3.1.3. TRPV1/TRPV2 Channels

TPR channels play a central role in intracellular Ca^2+^ dynamics and contribute to activity-dependent modulation of neuronal excitability.

CBD interacts with transient receptor potential vanilloid channels, particularly TRPV1 and TRPV2, which play critical roles in intracellular Ca^2+^ signaling and neuronal excitability. CBD transiently activates TRPV1, followed by rapid channel desensitization, a process that ultimately limits sustained Ca^2+^ influx during neuronal activation. Furthermore, TRPV2 is highly expressed in hippocampal neurons and has been proposed as a functionally relevant CBD-responsive channel involved in Ca^2+^ homeostasis, cellular stress responses, and neuroprotective signaling [7,10].

In the context of epilepsy, Ca^2+^ dysregulation represents a multilevel pathological process rather than a single molecular event. At the intrinsic cellular level, abnormal Ca^2+^ influx and impaired endoplasmic reticulum or mitochondrial Ca^2+^ handling promote excitotoxic stress and destabilize neuronal homeostasis. At the circuit level, these abnormalities facilitate Ca^2+^-dependent neurotransmitter release, hypersynchronous firing, and persistent excitation-inhibition imbalance. Extrinsic factors, including inflammatory mediators, hypoxia/ischemia, and imbalances in extracellular glutamate or adenosine, may further amplify Ca^2+^ instability within epileptic networks.

#### 3.1.4. ENT1-Adenosin Axis

The ENT1-adenosine pathway represents a key mediator of synaptic inhibition and activity-dependent suppression of excitatory neurotransmission.

CBD modulates purinergic signaling through inhibition of equilibrative nucleoside transporter 1 (ENT1), a key regulator of extracellular adenosine levels. By limiting adenosine reuptake, CBD increases extracellular adenosine concentrations and enhances adenosine A1 receptor-mediated inhibitory signaling. Because adenosine functions as an endogenous anticonvulsant neuromodulator, ENT1 inhibition represents an important mechanism linking CBD to the activity-dependent regulation of excitatory synaptic transmission [9,10].

#### 3.1.5. 5-HT1A and PPARγ Signaling

These pathways are primarily involved in neuroprotection, stress-response signaling and the regulation of neuroinflammatory processes.

CBD also modulates several signaling pathways involved in neuroprotection and stress responses. It acts as a partial agonist and positive allosteric modulator of 5-HT1A receptors, enhancing Gi/o protein-coupled inhibitory signaling that influences anxiety-related circuits and stress-responsive neuronal pathways [10]. Furthermore, CBD activates peroxisome proliferator-activated receptor-γ (PPARγ), a nuclear receptor that regulates inflammatory gene expression, oxidative stress responses, and metabolic homeostasis. Through PPARγ activation, CBD may contribute to the regulation of neuroinflammation associated with epileptogenesis [7,10].

Together, these pathways extend the mechanistic scope of CBD beyond synaptic modulation to include coordinated regulation of neuroprotective and stress-responsive signaling networks. Importantly, these upstream signaling pathways do not operate in isolation but converge on shared downstream effectors that directly regulate neuronal excitability.

#### 3.1.6. Ion Channel Modulation: Integrative Mechanism

Voltage-gated ion channels represent downstream effectors of neuronal excitability and membrane stability, integrating multiple upstream signaling pathways.

CBD interacts with multiple voltage-gated ion channels that regulate neuronal excitability. These include voltage-gated Na^+^ channels, T-type Ca^2+^ channels, selected K^+^ channels, and glycine receptors. While these targets overlap with certain conventional ASMs, CBD’s binding sites and functional modulation typically differ from those of classical channel blockers. Moreover, these interactions are considered components of CBD’s broader neuromodulatory network rather than primary standalone mechanisms, contributing to membrane stabilization across diverse hyperexcitable states.

Through these interactions, CBD may contribute to membrane stabilization and modulation of synaptic transmission, although the quantitative contribution of each channel type to anticonvulsant activity remains under investigation [10,11,12].

Thus, voltage-gated ion channel modulation may be best understood as an integrative downstream mechanism through which diverse upstream targets of CBD converge to stabilize neuronal excitability.

### 3.2. Network-Level Effects

Beyond individual molecular targets, these mechanisms converge at the level of neuronal circuits to regulate excitation-inhibition (E/I) balance and network stability.

In experimental studies of temporal lobe epilepsy, CBD modulates hippocampal network activity, reducing excitatory synaptic coupling among pyramidal neurons while preserving inhibitory interneuron function, including parvalbumin- and cholecystokinin-expressing interneurons [13].

Through the integration of these mechanisms, CBD suppresses pathological network synchronization and seizure propagation, providing mechanistic support for the clinical efficacy of purified CBD formulations (Epidiolex^®^/EPIDYOLEX^®^) as adjunctive therapies for refractory epilepsy [9].

Importantly, these effects should not be interpreted as independent processes, but rather as components of an integrated regulatory network that collectively stabilizes epileptic circuitry.

These network-level effects provide a functional framework linking the diverse molecular targets of CBD to the large-scale regulation of epileptic circuitry.

## 4. Mechanistic and Preclinical Evidence for Antiepileptic Effects of CBD

CBD provides a mechanistically distinct therapeutic strategy for drug-resistant epilepsy, supported by a growing body of experimental and translational evidence. The comparative routes of action between CBD and conventional ASMs are distinguished by their functional breadth. While conventional antiseizure medications (ASMs) typically target specific, well-defined molecular sites to suppress ictogenesis—such as voltage-gated sodium channel blockers (e.g., carbamazepine, phenytoin), GABAergic receptors (e.g., valproate, phenobarbital), and synaptic vesicle protein 2A (SV2A) (e.g., topiramate, levetiracetam, lamotrigine)—CBD’s efficacy is derived from a broader, polypharmacological profile. Unlike these classical agents that focus primarily on arresting electrical hypersynchrony, CBD utilizes multiple converging pathways involved in neuronal excitability, synaptic transmission, neuroinflammation, and metabolic homeostasis. This lack of significant target overlap with conventional ASMs suggests that CBD may provide synergistic benefits when used as an adjunctive therapy, addressing epileptogenic processes that remain refractory to standard ion channel-modulating drugs.

### 4.1. Evidence from Preclinical Models

Experimental studies in various epilepsy models provide functional validation for the molecular targets described in Section 3. For instance, in pentylenetetrazol (PTZ)-induced and pilocarpine-induced rodent seizure models [14,15,16], CBD significantly increased seizure thresholds and reduced excitatory synaptic transmission by modulating GPR55 signaling and facilitating activity-dependent desensitization of TRPV1 channels [17,18,19].

Furthermore, using hippocampal slice preparations, researchers have demonstrated that CBD-mediated ENT1 inhibition leads to a measurable increase in extracellular adenosine, which suppresses hypersynchronous neuronal firing as measured by local field potentials (LFPs) [9,20,21,22,23].

### 4.2. Modulation of Neuroinflammation

Chronic neuroinflammation is increasingly recognized as a key contributor to epileptogenesis. Several experimental studies demonstrate that CBD can attenuate inflammatory signaling pathways in the central nervous system.

CBD has been shown to reduce microglial activation and the production of pro-inflammatory cytokines such as TNF-α and IL-1β in experimental epilepsy models [24,25,26,27,28]. These anti-inflammatory actions may contribute to stabilization of neuronal microenvironments and mitigation of seizure-associated neurotoxicity.

### 4.3. Oxidative Stress and Mitochondrial Protection

Oxidative stress and mitochondrial dysfunction are additional mechanisms implicated in seizure-induced neuronal injury. Experimental studies indicate that CBD can reduce reactive oxygen species (ROS) production and improve mitochondrial membrane stability, thereby limiting oxidative damage and metabolic stress in neurons [29,30,31].

Through these antioxidant and mitochondrial protective effects, CBD may contribute to long-term preservation of neuronal integrity during chronic epileptic activity.

### 4.4. Translational and Clinical Evidence

Accumulating clinical evidence increasingly supports the translational relevance of these mechanistic pathways. Long-term extension studies and multinational real-world cohorts demonstrate sustained seizure reduction and functional stabilization during chronic CBD therapy, consistent with a disease-network–modifying profile rather than purely symptomatic suppression.

In developmental epileptic encephalopathies such as Dravet and Lennox–Gastaut syndrome, responder rates (≥50% seizure reduction) typically range from 40 to 60% during multi-year follow-up, with evidence of cumulative benefit suggestive of progressive attenuation of hyperexcitable networks and inflammatory burden [32,33].

CBD also demonstrates broad-spectrum efficacy in heterogeneous drug-resistant epilepsies, including non-syndromic developmental epileptic encephalopathies (DEEs), where clinically meaningful seizure reduction and occasional seizure freedom have been reported [34,35].

In contrast, adult focal epilepsies show more modest average response rates (approximately 25–40%), although emerging real-world data suggest the presence of biologically defined responder subgroups that may benefit from precision treatment potential [36].

Recent real-world evidence and long-term clinical outcomes across epilepsy syndromes through 2025 are summarized in Table 1.

Collectively, these findings indicate that CBD’s interactions with non-canonical receptors and transporters translate into functional suppression of hyperexcitable neuronal networks, supporting its anticonvulsant profile in both experimental and clinical contexts. Accordingly, whereas conventional ASMs primarily suppress seizure expression through relatively discrete molecular targets, CBD may additionally modulate upstream epileptogenic mechanisms at the cellular, network, inflammatory, and metabolic levels.

The principal mechanistic distinctions between conventional ASMs and CBD are summarized in Supplementary Table S2.

## 5. Advantages and Limitations of CBD

### 5.1. Approved Indications and Clinical Efficacy

Among various cannabinoid-based products, a highly purified, pharmaceutical-grade CBD oral solution is the most relevant formulation in the current therapeutic strategy. This purified form allows for consistent dosing and minimizes the unpredictable effects associated with non-standardized artisanal extracts. Currently, this formulation is approved as an adjunctive antiseizure therapy for Lennox–Gastaut syndrome (LGS), Dravet syndrome (DS), and tuberous sclerosis complex (TSC) in patients aged ≥ 2 years. CBD is administered in combination with conventional antiseizure medications, most commonly clobazam.

Multiple randomized controlled trials have demonstrated clinically meaningful efficacy of CBD in pediatric patients with LGS and DS. Across pivotal trials, CBD achieved median reductions in drop or convulsive seizure frequency of approximately 35–40%, compared with 15–20% in placebo groups, with significantly higher ≥50% responder rates, confirming its therapeutic benefit in severe developmental and epileptic encephalopathies [4].

In contrast, the efficacy of CBD appears to be syndrome- and age-dependent. In adults with drug-resistant focal epilepsy, a large randomized, double-blind, placebo-controlled trial evaluating transdermal CBD (195 mg or 390 mg twice daily) failed to demonstrate a statistically significant reduction in seizure frequency compared with placebo over a 12-week treatment period [3]. These findings suggest that the clinical benefit of CBD is robust in specific childhood-onset epileptic encephalopathies but remains unproven in adult focal epilepsies, underscoring the need for precision-based patient selection.

### 5.2. Advantages

#### 5.2.1. Disease-Modifying Potential

CBD exhibits therapeutic effects extending beyond symptomatic seizure suppression and may exert disease-modifying properties in developmental and epileptic encephalopathies. In DS and LGS, adjunctive CBD therapy results in sustained seizure reduction of approximately 30–50%, with ≥50% responder rates occurring two- to threefold more frequently than those obtained with placebo and a subset of patients achieving seizure freedom. These durable responses suggest that CBD may attenuate epileptogenic network remodeling rather than merely suppress ictal activity [37]. Unlike most conventional antiseizure medications that primarily suppress ictal activity through discrete ion-channel modulation, CBD exerts broader network-level effects and mitochondrial protection effects. These multimodal properties may provide therapeutic benefits in developmental epileptic encephalopathies where epileptogenesis involves complex molecular and circuit-level dysregulation.

#### 5.2.2. Anti-Inflammatory and Neuroprotective Effects

CBD exerts robust anti-inflammatory and neuroprotective actions, representing a therapeutic route that is largely unaddressed by conventional ASMs. By suppressing microglial activation and downregulating pro-inflammatory cytokines (including TNF-α and IL-1β), CBD mitigates the neurobiological substrate underlying chronic epilepsy. This neuroprotective route, combined with oxidative stress reduction and mitochondrial stabilization [38], contributes to long-term preservation of neuronal integrity, contrasting with the purely symptomatic suppression offered by most traditional medications. These multimodal effects mitigate seizure-induced neuronal injury and contribute to increased seizure thresholds, supporting a potential role for CBD in modifying the neurobiological substrate underlying epilepsy [39].

#### 5.2.3. Favorable Cognitive Tolerability

Unlike many conventional antiseizure medications, CBD lacks psychoactive properties and demonstrates minimal adverse effects on cognition, behavior, and psychomotor performance, particularly in pediatric populations. Long-term extension studies and caregiver-reported outcome measures have indicated stable or improved cognitive function and quality of life, supporting its favorable neurocognitive tolerability profile [40].

### 5.3. Limitations

#### 5.3.1. Drug–Drug Interaction

CBD is a potent inhibitor of cytochrome P450 enzymes, particularly CYP2C19 and CYP3A4, resulting in clinically significant drug–drug interactions. Co-administration with clobazam markedly increases plasma concentrations of its active metabolite N-desmethylclobazam, thereby enhancing sedative adverse effects and necessitating dose reduction [37,41]. Concomitant use with valproate is associated with an increased risk of hepatic enzyme elevation, requiring careful therapeutic drug monitoring and frequent dose adjustments.

#### 5.3.2. Hepatotoxicity Risk

Dose-dependent elevations in serum transaminases have been consistently reported in patients receiving CBD, particularly at higher doses and in combination with valproate [41,42]. Although most cases are reversible, this hepatotoxic potential necessitates regular liver function monitoring, increasing clinical burden and limiting the feasibility of CBD as a first-line antiseizure medication.

#### 5.3.3. Interindividual Variability

CBD demonstrates substantial interindividual variability in pharmacokinetics and therapeutic response. Variations in absorption, metabolism, and genetic polymorphisms in CYP enzymes contribute to unpredictable efficacy and tolerability. Furthermore, escalation to higher doses does not consistently yield proportional improvements in seizure control, indicating a plateau in dose–response relationships and complicating individualized dosing strategies [42,43].

#### 5.3.4. Regulatory and Formulation Constraints

Despite demonstrated efficacy in severe childhood-onset epilepsies, evidence supporting CBD use in focal epilepsy and adult populations remains limited. High treatment costs, restricted insurance coverage, and regulatory limitations substantially reduce accessibility [42]. In addition, formulation-related challenges, the need for strict monitoring of potential toxicities, and the complexity of dose adjustments in polytherapy settings further hinder its development and adoption as a primary anticonvulsant agent. Consequently, CBD is currently positioned primarily as an adjunctive rather than a first-line antiseizure therapy.

These limitations underscore that CBD is currently best positioned as an adjunctive therapy rather than a universal replacement for conventional antiseizure medications. Identification of responder populations and optimized treatment windows will be essential for improving its clinical utility.

## 6. Pediatric Epilepsy and Critical Therapeutic Windows

Pediatric epilepsies represent a unique therapeutic context in which early and effective seizure control may exert long-term disease-modifying effects [44]. CBD has attracted increasing interest as a potential therapeutic option in early-life epilepsy. Notably, CBD has demonstrated efficacy in several severe childhood-onset epileptic syndromes [45,46,47].

However, despite its growing clinical use, systematic data regarding optimal timing of CBD intervention, long-term developmental outcomes, and its role across broader pediatric epilepsy populations remain limited [48,49]. Similar gaps exist for special physiological conditions such as pregnancy, where seizure control must be balanced against fetal safety and neurodevelopmental considerations [50]. These unmet clinical needs highlight the importance of understanding how therapeutic windows during brain maturation may influence both treatment responsiveness and long-term neurological trajectories.

### 6.1. Developmental Basis for Superior Treatment Responsiveness in Children

Pediatric epilepsies exhibit fundamentally distinct therapeutic responsiveness compared with adult-onset epilepsy because epileptic activity directly interferes with ongoing brain maturation. In early life, seizures are not merely transient manifestations of abnormal neuronal firing but actively remodel synaptic architecture, neuronal connectivity, and network excitability. Consequently, effective seizure suppression during critical developmental stages can normalize aberrant circuit formation, thereby exerting disease-modifying effects rather than simply reducing seizure frequency.

This heightened developmental plasticity underlies the superior treatment responsiveness observed in pediatric epilepsy, in which timely pharmacological intervention can permanently alter epileptogenic trajectories and prevent lifelong neurodevelopmental disability. Conversely, delayed or insufficient seizure control allows pathological network remodeling to stabilize, resulting in persistent epilepsy and progressive cognitive impairment.

### 6.2. GABAergic Immaturity and Early-Life Epileptogenesis

A major neurobiological mechanism contributing to early-life epileptogenesis is the immaturity of GABAergic neurotransmission. In the neonatal and infant brain, γ-aminobutyric acid (GABA) exerts depolarizing and excitatory effects due to elevated intracellular chloride concentrations mediated by high expression of the Na^+^–K^+^–2Cl^−^ cotransporter 1 (NKCC1) and reduced expression of the K^+^–Cl^−^ cotransporter 2 (KCC2). This excitatory GABAergic tone enhances network hyperexcitability and facilitates epileptiform synchronization.

Recurrent seizures during this vulnerable developmental period further reinforce aberrant synaptic wiring and excitatory–inhibitory imbalance, thereby accelerating the consolidation of epileptogenic circuits. Failure to interrupt this pathological process promotes progressive pharmacoresistance and irreversible neurodevelopmental impairment.

### 6.3. Developmental Timing of Seizure Control and Potential Disease Modification

The first three years of life constitute a highly dynamic neurodevelopmental period marked by intense synaptogenesis, activity-dependent synaptic pruning, and progressive myelination. During this period, neuronal circuits exhibit exceptional plasticity while remaining highly vulnerable to maladaptive remodeling triggered by recurrent epileptic activity [51]. Inadequate seizure control during this critical phase promotes structural consolidation of hyperexcitable networks, embedding pathological activity within developing neural architecture and transforming episodic seizures into a persistent network disorder.

This phenomenon is consistently observed across a broad spectrum of developmental epileptic encephalopathies (DEEs), including Dravet syndrome, West syndrome, and epilepsies associated with SCN1A, KCNQ2-, CDKL5, PCDH19, STXBP1, and WWOX mutations [52,53]. Across these genetically diverse conditions, delayed initiation of effective seizure suppression is strongly associated with severe cognitive impairment, autism spectrum disorder, progressive neurodevelopmental disorder, and the emergence of pharmacoresistant epilepsy. Collectively, these clinical observations support the concept that early and sustained seizure control during vulnerable developmental periods may exert disease-modifying effects by limiting aberrant circuit maturation and maladaptive network stabilization.

Complementing these clinical data, mechanistic studies have identified dysregulated mTOR signaling as a central driver of epileptogenesis and pathological network remodeling in multiple neurodevelopmental epilepsy models. In mTORopathy-related conditions, including tuberous sclerosis complex and DEPDC5-associated focal cortical dysplasia, pharmacological inhibition of mTOR activity suppresses seizure development, normalizes synaptic organization, and stabilizes hyperexcitable circuits in preclinical systems [54]. Despite these promising findings, translation into broader clinical practice remains limited, as mTOR-targeted therapies have not been formally approved for acquired epilepsies or non-TSC genetic mTORopathies [55,56]. Currently, everolimus constitutes the sole clinically approved mTOR inhibitor, indicated specifically for treatment-resistant seizures associated with tuberous sclerosis complex [57].

Taken together, these converging clinical and experimental data indicate that developmental epilepsies are governed by narrow, syndrome-specific therapeutic windows during which aberrant network remodeling remains biologically modifiable. Beyond these periods, epileptic circuits progressively stabilize into entrenched pharmacological configurations that exhibit reduced responsiveness to pharmacological intervention. Therapeutic strategies that leverage heightened developmental plasticity therefore hold particular promise for limiting irreversible network pathology and improving long-term neurological outcomes, as summarized across developmental stages in Table 2.

### 6.4. Unresolved Risks and Safety Considerations of CBD in Pediatric Epilepsy

Despite its demonstrated efficacy in several severe childhood epileptic encephalopathies, important safety considerations remain when CBD is used in pediatric populations. Long-term neurodevelopmental safety remains insufficiently defined, particularly regarding potential effects on cognitive maturation and neural circuit development. In addition, CBD therapy carries a notable clinical monitoring burden due to dose-dependent elevations of hepatic transaminases, particularly when co-administered with valproate. Critical drug–drug interactions mediated through cytochrome P450 enzymes, most notably the inhibition of CYP2C19, which leads to potentially toxic levels of N-desmethylclobazam, further complicate pharmacological management.

Moreover, current evidence supports CBD efficacy primarily in specific developmental epileptic encephalopathies such as Dravet syndrome, Lennox–Gastaut syndrome, and tuberous sclerosis complex, rather than across the full spectrum of pediatric epilepsies. Considerable interindividual variability in treatment response also suggests the presence of biologically distinct responder populations. These unresolved issues highlight the need for continued mechanistic studies and longitudinal clinical investigations to better define the developmental safety profile and optimal therapeutic positioning of CBD in pediatric epilepsy.

## 7. Epilepsy in Pregnancy: Balancing Seizure Control and Teratogenic Risk

### 7.1. Clinical Dilemma: Seizure Suppression vs. Fetal Safety

Epilepsy during pregnancy presents a complex therapeutic challenge in which effective seizure control must be balanced against medication-related teratogenic risk. Uncontrolled maternal seizures substantially increase the risk of hypoxia, physical trauma, cerebrovascular events, disseminated intravascular coagulation, postpartum hemorrhage, and maternal mortality, and are associated with elevated rates of psychiatric morbidity and long-term maternal complications [58,59].

From the fetal perspective, maternal epilepsy is linked to increased risks of stillbirth, preterm delivery, low birth weight, neonatal mortality, and reduced Apgar scores [58,59,60]. Importantly, seizure recurrence itself constitutes an independent predictor of adverse perinatal outcomes, underscoring the necessity of pharmacological seizure suppression despite potential fetal exposure. Consequently, epilepsy management during pregnancy inherently involves a trade-off between seizure prevention and teratogenic risk, highlighting the need for evidence-based optimization of therapeutic strategies.

### 7.2. Teratogenic Profiles of Antiseizure Medications

**Figure 2:**
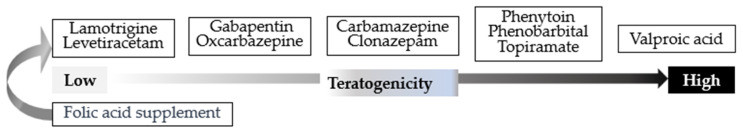
Relative teratogenic risk spectrum of commonly used antiseizure medications during pregnancy.

Conventional antiseizure medications (ASMs) exhibit marked interdrug variability in teratogenic potential. The prevalence of major congenital malformations (MCMs) among ASM-exposed pregnancies ranges from approximately 2% to 8%, exceeding the baseline population risk of 1–2% [61,62]. Teratogenicity varies according to drug class, dosage, and treatment regimen. These relative differences in teratogenic risk across commonly used ASMs are summarized in Figure 2.

Among commonly prescribed agents, valproic acid confers the highest teratogenic burden, with MCM rates approaching 10% and strong associations with neural tube defects, congenital heart disease, craniofacial abnormalities, and orofacial clefts [61,62]. Prenatal exposure has also been consistently linked to long-term neurodevelopmental impairments, including cognitive deficits, behavioral disturbances, and increased risk of autism spectrum disorders [63]. Accordingly, international guidelines strongly discourage its use in women of childbearing potential unless no effective alternative exists [64,65].

Teratogenic risk is further amplified by polytherapy and high-dose exposure, which are associated with significantly higher rates of congenital malformations, stillbirth, intrauterine growth restriction, and preterm birth compared with monotherapy [61,62]. Contemporary clinical practice therefore prioritizes monotherapy at the lowest effective dose to optimize seizure control while minimizing fetal risk [66,67,68].

Antiseizure medications are positioned along a continuum of increasing teratogenicity, with lamotrigine and levetiracetam demonstrating the most favorable reproductive safety profiles, and valproic acid associated with the highest risk of major congenital malformations. Periconceptional folic acid supplementation is depicted as a protective modifier across exposure groups [64,65,66,69].

### 7.3. Evidence Gaps and the Need for Mechanistic Modeling

Despite advances in pregnancy registries and pharmacovigilance, critical gaps persist in understanding ASM-specific neurodevelopmental toxicity, dose–response relationships, gene–drug interactions, and periods of heightened fetal vulnerability. Available data remain predominantly observational and limited by confounding and heterogeneity in seizure phenotypes.

Notably, robust human data regarding congenital and long-term neurodevelopmental outcomes following in utero exposure to prescription cannabidiol are lacking, with existing registries providing little systematically analyzed CBD-specific evidence [70]. Consequently, current guidelines rely largely on population-level risk estimates and remain insufficient for individualized maternal–fetal risk stratification.

## 8. Brain Organoids as Translational Models of Epilepsy

Human brain organoids have emerged as advanced three-dimensional (3D) experimental platforms for modeling epilepsy, particularly genetic and developmental epileptic encephalopathies. By recapitulating key aspects of early human cortical development, synaptogenesis, and network maturation, organoids enable mechanistic interrogation of epileptogenesis that is not feasible in conventional two-dimensional (2D) cultures or animal models. The use of patient-derived induced pluripotent stem cells (iPSCs) and CRISPR-engineered isogenic lines further allows direct linkage of pathogenic genotypes to network-level phenotypes, facilitating quantitative analysis of hyperexcitability, seizure propagation, and precision drug screening [71,72,73].

**Figure 3:**
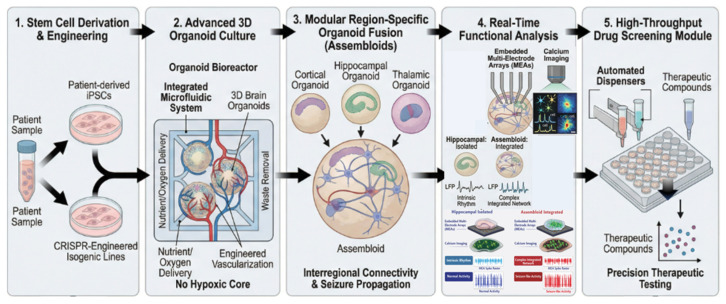
Ideal Human Brain Organoid Platform for Epilepsy Modeling. This schematic illustrates the integrated pipeline for human-specific epilepsy research. (1) Patient-derived iPSCs and CRISPR-engineered lines provide a genetic basis for disease modeling. (2) Advanced 3D culture systems with microfluidics ensure physiological viability. (3) Region-specific assembloids enable the study of interregional connectivity and seizure propagation. (4) Real-time functional analysis using embedded MEAs and calcium imaging enables the precise characterization of network-level hyperexcitability; differentiation of normal activity (sparse, asynchronous firing) from seizure-like patterns (dense, synchronized burst firing, increased spike frequency and amplitude). (5) The high-throughput module facilitates precision therapeutic testing of compounds like CBD, bridging the gap between preclinical animal data and human clinical application.

Figure 3 illustrates these key concepts in an integrated human brain organoid platform for epilepsy modeling, which is further elaborated in the following sections.

### 8.1. Genetic Epilepsy Organoids

Organoids generated from patients with monogenic epilepsies reproducibly exhibit disease-relevant neurodevelopmental abnormalities that mirror human developmental and epileptic encephalopathies (DEEs). For example, organoids derived from individuals with WOREE syndrome caused by WWOX mutations demonstrate disrupted cortical lamination, excessive neural progenitor proliferation, and impaired neuronal migration. *WWOX*-deficient organoids further exhibit imbalanced neural population dynamics, including upregulation of GABAergic markers such as GAD1 and impaired neuronal differentiation, recapitulating key pathological features observed in severe DEEs [71,72]. Similarly, organoids modeling Miller–Dieker syndrome display lissencephaly-like structural disorganization and defective radial migration, providing additional validation of the fidelity of organoid-based disease modeling.

### 8.2. Modeling Network Hyperexcitability

Patient-derived epilepsy organoids reproducibly exhibit intrinsic network hyperexcitability that can be quantified using calcium imaging, multielectrode array recordings, and patch-clamp electrophysiology. In Angelman syndrome organoids, loss of *UBE3A* promotes network hypersynchrony through dysregulation of large-conductance calcium-activated potassium (BK) channels, resulting in spontaneous epileptiform activity.

*WWOX*-deficient organoids exhibit epileptiform local field potentials, increased oscillatory power at low frequencies, and hypersynchronous discharges following 4-aminopyridine challenge, reflecting a profound imbalance between excitatory and inhibitory network activity [71,72,74].

### 8.3. Modeling Seizure Propagation and Network Synchronization

Beyond local hyperexcitability, organoid platforms enable investigation of seizure propagation and large-scale network synchronization. Organoid slice cultures and regionally fused “assembloids” recapitulate interregional connectivity and permit dynamic analysis of seizure propagation. These models display δ-high-frequency oscillation (HFO) coupling and recurrent high-frequency oscillations reminiscent of human seizure substates.

In Rett syndrome fusion organoids, hypersynchronous epileptiform spikes propagate across fused cortical regions, revealing human-specific circuit vulnerabilities that are not fully reproduced in rodent models [71,72,74].

### 8.4. Drug Screening and Precision Therapeutic Modeling

Brain organoids provide scalable platforms for high-content and high-throughput antiepileptic drug screening and precision therapeutic modeling. In *WWOX*-deficient organoids, ectopic *WWOX* expression rescues hyperexcitability and neuronal differentiation defects, demonstrating the reversibility of disease phenotypes. Pharmacological modulation of BK channels attenuates network hyperactivity, while inhibition of aberrant Wnt signaling corrects excessive progenitor proliferation in related models.

Collectively, these findings establish brain organoids as powerful translational platforms for personalized antiseizure drug testing, mechanistic target validation, and preclinical screening of next-generation therapies [71,72].

## 9. Cannabidiol in Human Brain Organoids: Research Findings

### 9.1. Validation of CBD’s Antiseizure and Neuroprotective Effects

CBD exerts broad-spectrum antiseizure and neuroprotective effects; however, direct validation on developing human cortical networks remained limited until the advent of human brain organoid platforms. Human cerebral organoids overcome this limitation by recapitulating early cortical cytoarchitecture and synchronized neuronal network activity, thereby enabling direct interrogation of CBD effects within a human-specific developmental context.

Using oxygen–glucose deprivation (OGD)-induced hyperexcitability paradigms, organoid-based electrophysiological studies have demonstrated that CBD robustly suppresses pathological network synchronization and reduces neuronal firing rates. Specifically, CBD prevents OGD-induced increases in power spectral density, with efficacy comparable to bumetanide, confirming direct normalization of aberrant network oscillations [75].

Furthermore, CBD markedly attenuates oxidative stress and glutamate-mediated excitotoxicity more effectively than classical antioxidants, highlighting its intrinsic antioxidant and neuroprotective capacity in human cortical-like tissues [75,76,77]. Collectively, these findings provide human-specific validation that CBD can normalize neuronal network hyperexcitability and mitigate redox imbalance in developing cortical circuits.

### 9.2. Impact on Human Neuronal Maturation and Genotype-Specific Responses

Beyond acute seizure suppression, patient-derived organoids have provided critical genotype-specific insights into CBD responsiveness. In an organoid model of Dravet syndrome and WWOX deficiency (WOREE syndrome), CBD treatment restored interneuron-mediated inhibitory tone and significantly reduced spontaneous epileptiform discharges and high-frequency oscillations (HFOs). These organoid models also provide mechanistic insight into how CBD may influence human neurodevelopment. Transcriptomic profiling suggests that CBD may promote GABAergic maturation by modulating the expression of chloride transporters NKCC1 and KCC2, thereby facilitating the developmental shift toward inhibitory network stabilization during early cortical development [75]. This maturation-promoting effect is particularly relevant to developmental epileptic encephalopathies, in which delayed GABA polarity transitions contribute to network hyperexcitability.

Importantly, organoid studies also reveal substantial variability in functional responses to CBD exposure, indicating intrinsic interindividual differences in cannabinoid sensitivity. Experimental studies have shown that low micromolar concentrations of CBD can modulate neuronal activity without inducing cytotoxicity; however, functional outcomes vary markedly between organoids. Such heterogeneity may arise from variability in endocannabinoid receptor expression, genetic background, or developmental stage of neural networks [76,77,78].

Rather than acting through a single receptor pathway, accumulating evidence supports a polypharmacological model in which CBD coordinately modulates multiple signaling systems relevant to excitation-inhibition (E/I) balance [9,75,79]. These findings provide a potential molecular basis for the observed responder-non-responder heterogeneity observed in clinical settings and highlight the potential of organoid platforms for identifying precision medicine strategies in epilepsy.

## 10. Methodological Frameworks for CBD-Organoid Research

To systematically investigate the mechanisms and therapeutic responses described above, recent studies have established diverse organoid-based experimental platforms integrating disease modeling, multimodal electrophysiology, and developmental stage-specific analyses.

### 10.1. Genetic and Acquired Hyperexcitability Models

Human brain organoids have emerged as advanced three-dimensional (3D) in vitro platforms that recapitulate key aspects of human cortical development, including neuronal migration, cortical lamination, and spontaneous network activity. Since 2019, the application of cerebral organoids and multi-regional assembloids to epilepsy research has expanded rapidly, particularly for modeling developmental and genetic epileptic encephalopathies (DEEs) and acquired neonatal seizure models [71,72,75].

These patient-specific iPSC-derived or CRISPR-engineered organoids can reproduce key pathological features of DEEs, including disrupted cortical organization, aberrant neuronal synchronization, and spontaneous epileptiform discharges, thereby providing human-specific developmental contexts that are incompletely modeled in animal systems.

Genetic epilepsy models currently include Rett syndrome, Dravet syndrome, WWOX-related epileptic encephalopathy (WOREE), protocadherin-19 (PCDH19)–related epilepsy, and SCN1A/SCN8A channelopathies. WWOX knockout and patient-derived organoids exhibit cortical disorganization, altered excitatory-inhibitory balance, and spontaneous epileptiform oscillations, whereas Rett and Angelman syndrome organoids display hypersynchronous network activity associated with impaired inhibitory maturation and ion channel dysregulation [71,72,74,80].

In addition to genetic models, acquired hyperexcitability paradigms have been using oxygen–glucose deprivation (OGD) or pharmacological and chemogenic induction of hyperexcitability. OGD-treated cortical organoids exhibit chloride imbalance, delayed GABAergic maturation, and spontaneous epileptiform activity, thereby modeling hypoxic–ischemic neonatal seizure mechanisms [71,81,82].

Recent methodological advances further integrate these models with CRISPR-engineered isogenic lines and regionally patterned assembloid systems. In particular, cortical–ganglionic eminence assembloids enable the modeling of interneuron migration and circuit integration, providing a platform to investigate how pharmacological agents such as CBD may stabilize hypersynchronous human neural networks. By combining these strategies, organoid platforms offer a versatile experimental framework for human-specific epilepsy modeling and precision drug testing.

### 10.2. Multimodal Analytical Platforms for Drug Screening

Methodologically, the precision evaluation of CBD within organoid systems relies on the integration of high-resolution electrophysiological, calcium imaging, and transcriptomic platforms to quantify network-level phenotypes and drug responses. Current research frameworks employ patch-clamp recordings, local field potential (LFP) measurements, and multielectrode array (MEA) systems, which are essential for detecting shifts in firing rate modulation, oscillatory synchronization and spontaneous epileptiform discharges following CBD treatment, providing a functional readout of its anticonvulsant activity in a 3D human environment [75,83].

Complementary calcium (Ca^2+^) imaging enables high-throughput visualization of population-level network dynamics. This platform is particularly effective for characterizing hypersynchronization and seizure-like bursts in DEE and OGD-induced models, allowing for real-time monitoring of calcium transient normalization as a primary endpoint for pharmacological interventions [74,75].

Furthermore, transcriptomic profiling, encompassing both bulk and single-cell RNA sequencing (scRNA-seq), is utilized to identify the molecular pathways influenced by CBD. This includes the analysis of chloride transporter expression, oxidative stress–response genes, and synaptic development programs [75,83]. The integration of these multi-omics datasets with functional assays enables the correlation of genetic background–specific vulnerabilities with individualized drug responses, reinforcing the role of cerebral organoids as robust precision medicine platforms for validating next-generation CBD-based therapeutics.

### 10.3. Developmental Stage-Specific Modeling of Epilepsy

Human cerebral organoids enable epilepsy-relevant developmental windows to be modeled in vitro by recapitulating the chronological stages of human brain maturation. As summarized in Table 3, these stage proxies allow researchers to investigate the efficacy of CBD across distinct neurodevelopmental milestones.

During the prenatal stage (culture days 60–100), corresponding to early molecular pathogenesis, organoids serve as a platform for presymptomatic modeling of genetic epilepsies like *SCN1A* and *KCNQ2* [84]. As maturation progresses to the neonatal or perinatal-like stage (culture days 120–200), emergence of the GABAergic excitatory-to-inhibitory switch and initial network bursts provides a critical window for studying the onset of neonatal developmental and epileptic encephalopathies, such as *KCNQ2*- and *WWOX*-associated DEE [85,86,87].

In more mature cultures, specifically the early postnatal or child-like stage (culture days 250–350), organoids exhibit theta-gamma-band oscillations and increasing astrocytic ramification [85,86]. This stage parallels the clinical period when syndromes such as Dravet syndrome and West syndrome commonly manifest. Finally, long-term cultured organoids (>400 days), representing the late-stage neural maturation equivalent of childhood or pre-adolescence, facilitate the investigation of chronic epilepsy phenotypes and post-critical window circuit alterations [85,86].

## 11. Limitations of Brain Organoid Models in Translational Epilepsy Research

Despite the growing enthusiasm for brain organoid platforms as personalized disease models and drug-testing tools, particularly patient-derived organoids (PDOs), their design, fabrication, and long-term maintenance remain technically demanding and highly resource-intensive, creating substantial barriers to scalability, reproducibility, and standardization across laboratories, thereby constraining their translational reliability.

A major concern is the pronounced organoid-to-organoid variability observed even within identical cell lines and experimental protocols. This batch-dependent heterogeneity compromises experimental consistency and restricts the capacity of organoids to reliably predict individualized therapeutic responses. The lack of standardized differentiation pipelines further exacerbates inter-study variability, hindering large-scale validation and regulatory translation.

Critical physiological features essential for accurate disease modeling and pharmacological assessment are also incompletely represented. Most brain organoids lack functional vascular networks, resulting in non-physiological nutrient and oxygen gradients and altered drug exposure profiles. In particular, the technical optimization required for precise pharmacological assays in 3D platforms remains insufficient; standardized protocols for the administration and dose–response monitoring of lipophilic compounds, such as Cannabidiol (CBD), have not been fully established. In addition, immature or aberrant cytochrome P450 enzyme expression distorts metabolic responses compared with in vivo human brain conditions. This lack of methodological maturation, combined with the structural complexity of organoids, complicates the direct translation of CBD’s antiseizure mechanisms from conventional 2D cultures or animal models to human-centric organoid systems. The absence of microglia and broader immune components further limits the modeling of neuroinflammatory mechanisms increasingly implicated in epileptogenesis and treatment responsiveness.

Importantly, current organoid systems largely recapitulate early developmental brain stages, corresponding to fetal or neonatal stages. This developmental immaturity restricts the investigation of mature network dynamics, synaptic stabilization, and long-term disease progression. In particular, hippocampal organoids—central to temporal lobe epilepsy research—remain insufficiently optimized in cytoarchitecture, connectivity, and functional circuit organization, constraining their mechanistic and translational utility.

Finally, the incomplete reconstruction of blood–brain barrier structure and function further limits accurate prediction of central nervous system drug penetration and toxicity. Collectively, these limitations underscore that while brain organoids provide powerful insights into neurodevelopmental and epileptogenic processes, they currently fall short as standalone predictive platforms for individualized therapeutic decision-making in translational epilepsy research. This technical gap explains the current scarcity of direct literature regarding CBD’s efficacy and its mechanistic comparison with conventional ASMs in organoid-based epilepsy models, emphasizing that this is an emerging field requiring further methodological validation.

Furthermore, several mechanistic pathways discussed in this review—including modulation of excitatory-inhibitory balance, intracellular Ca^2+^ signaling, and neuroinflammatory regulation—are not yet fully reproduced in current organoid systems. In particular, incomplete maturation of inhibitory interneuron networks and limited long-range circuit connectivity constrain the ability of organoids to model seizure propagation and hypersynchronous network activity observed in human epilepsy. These developmental and circuit-level limitations may partially explain why translating the molecular mechanism of CBD into measurable network-level outcomes remains technically challenging in organoid platforms.

In addition, pharmacological evaluation of CBD within organoid systems presents unique methodological challenges. CBD is a highly lipophilic compound whose pharmacokinetics in vivo depend on blood–brain barrier permeability, systemic metabolism, and protein binding. These physiological determinants are not fully recapitulated in organoid cultures, potentially altering drug distribution and effective tissue concentrations. Consequently, caution is required when extrapolating CBD dose–response relationships observed in organoid experiments to clinical contexts.

## 12. Future Perspective: Human Experimental Platforms for Antiseizure Drug Safety and Cannabidiol Mechanisms

Despite advances in pregnancy registries and observational pharmacovigilance, clinical gaps persist in understanding antiseizure-medication-related neurodevelopmental toxicity, dose–response relationships, gene-drug interactions, and sensitive developmental windows during fetal brain maturation. Current evidence is derived predominantly from observational studies and remains limited by confounding factors, heterogeneous epilepsy phenotypes, and incomplete mechanistic resolution.

Emerging human-relevant experimental platforms, including stem cell-derived brain organoids and developmental neurotoxicity assays, offer complementary preclinical systems for investigating ASM-associated neurodevelopmental risk across distinct developmental windows. Rather than serving as predictive clinical tools at present, these models function primarily as exploratory systems capable of interrogating stage–specific drug effects on neuronal differentiation, circuit maturation, and network excitability within a human developmental context.

Importantly, only a limited number of studies have directly examined the effects of CBD in brain organoid-based epilepsy models, indicating that this field remains largely exploratory and methodologically underdeveloped.

**Figure 4:**
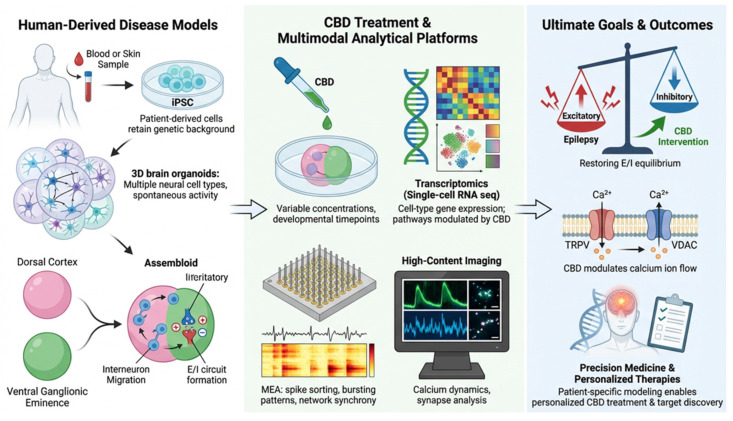
Organoid-based experimental framework for investigating cannabidiol (CBD) effects in developmental epilepsy. The schematic illustrates a human-relevant research strategy integrating advanced organoid platforms and multimodal analysis. (**Left**) Human-Derived Disease Models include patient-specific induced pluripotent stem cell (iPSC)-derived organoids and cortical–ganglionic eminence assembloids that recapitulate developmental epileptic circuits. (**Middle**) Multimodal Analytical Platforms, including high-density multielectrode arrays (HD-MEA), high-content imaging and transcriptomic profiling, enable monitoring of neuronal network activity and molecular responses to CBD exposure. (**Right**) Ultimate Goals & Outcomes include CBD-mediated modulation of intracellular Ca^2+^ homeostasis, restoration of excitation–inhibition (E/I) balance, and the development of precision therapeutic strategies. Arrows indicate the direction of workflow progression and mechanistic interactions, while different colors represent distinct functional modules, including disease modeling, analytical platforms, and therapeutic outcomes.

As previously discussed, to clearly elucidate the complex pharmacological mechanisms of CBD, advanced human disease platforms—such as assembloids and vascularized organoids—are essential, moving beyond simplified cellular models. This integrated research strategy and the future direction of multimodal analytical platforms are illustrated in Figure 4.

Within this framework, although direct evidence of CBD efficacy in brain organoids is currently scarce due to the aforementioned technical hurdles, these models hold significant potential for bridging the gap between clinical observation and mechanistic understanding.

Specifically, our proposed strategy involves the use of patient-specific models to dissect how CBD uniquely regulates intracellular *Ca*^2+^ homeostasis and E/I balance—mechanisms that are often dysregulated at the intrinsic cellular and circuit levels in pediatric epilepsy. Future research should focus on optimizing the 3D microenvironment to facilitate precise CBD treatment and identifying stage-specific responses during neural maturation. The development of “assembloids”—integrating cortical and ganglionic eminence organoids—will be pivotal in modeling the migration of inhibitory interneurons and the subsequent effects of CBD on stabilizing hypersynchronous neural circuits. Such approaches may allow investigators to examine how CBD influences neuronal maturation, synaptic development, and network excitability within controlled human-derived experimental systems. Controlled manipulation of developmental stage, genetic background, and pharmacological exposure enables the investigation of cellular and circuit-level responses that are difficult to resolve in clinical cohorts alone.

Modeling early neurodevelopmental processes in vitro complements epidemiological pregnancy registry data by enabling mechanistic interrogation of drug exposure, though these models require extensive validation before any translational relevance can be inferred.

Integration of electrophysiological profiling, such as high-density multi-electrode arrays (MEAs) for monitoring real-time burst activity, high-content imaging, and transcriptomic analyses within organoid systems, may further refine our understanding of CBD-mediated cellular and network-level effects. By directly addressing the current scarcity of comparative studies, these multimodal datasets have the potential to advance mechanistic frameworks of antiseizure drug action—specifically by distinguishing CBD’s polypharmacological profile from conventional ASMs—and identify candidate biomarkers of therapeutic response in future research settings.

Collectively, these experimental modeling strategies may complement epidemiological evidence by advancing the mechanistic understanding of antiseizure medication safety during pregnancy and informing the long-term development of safer therapeutic approaches.

Future investigations should therefore prioritize several key methodological directions. First, the development of vascularized and immune-competent organoid systems incorporating microglia will be essential for modeling neuroinflammatory mechanisms relevant to epileptogenesis and CBD action. Second, improved pharmacokinetic modeling within organoid platforms, including controlled drug delivery systems and microfluidic perfusion environments, may allow more physiologically relevant assessment of lipophilic compounds such as CBD. Third, integration of patient-specific iPSC-derived organoids with multi-omics profiling and high-resolution electrophysiology may facilitate identification of mechanistically defined responder populations. These advances may ultimately enable organoid-based platforms to serve as translational bridges between molecular mechanisms, developmental neurobiology, and precision epilepsy therapeutics.

## 13. Conclusions

In summary, purified cannabidiol (CBD) has emerged as a clinically relevant adjunctive therapy for selected developmental and drug-resistant epilepsies, particularly in pediatric syndromes such as Dravet syndrome, Lennox–Gastaut syndrome, and tuberous sclerosis complex. Unlike conventional antiseizure medications (ASMs), which typically target discrete ion channels or synaptic pathways, CBD exerts anticonvulsant and neuroprotective effects through a broader polypharmacological network, including GPR55 antagonism, TRPV1 modulation, adenosine signaling, intracellular *Ca*^2+^ regulation, and attenuation of neuroinflammatory pathways.

Despite these therapeutic advantages, important limitations remain. Clinical response to CBD shows substantial interindividual variability, and significant drug–drug interactions with conventional ASMs require careful monitoring. Moreover, evidence regarding CBD exposure during pregnancy or early neurodevelopment remains limited, and its long-term developmental safety has not yet been fully established.

Although further methodological validation is required, the integration of organoid-based systems with multimodal electrophysiological and transcriptomic analyses may advance mechanistically informed and developmentally safe epilepsy therapies in the future. Accordingly, the future value of CBD in epilepsy will depend not only on broader clinical adoption but also on identifying mechanistically defined responder populations and developmentally appropriate treatment windows through integrated human-specific translational platforms.

## Figures and Tables

**Table 1:** Comparative Real-World and Long-Term Clinical Response to Cannabidiol Across Epilepsy Syndromes and Developmental Stages.

Syndrome/ Population	Typical Age/Developmental Stage at Onset	≥50% Responder Rate (%)	Seizure Freedom (%)	Long-Term Trends & Notes
Dravet syndrome (DS)	Infancy and early childhood; highly immature, plastic networks	~60	Low ^†^	Retrospective multicenter data show ~45–50% at 12 mo.; sustained reduction over time. Clobazam co-treatment did not significantly alter responder rates
Lennox–Gastaut syndrome (LGS)	Early childhood; diffuse network pathology and severe developmental impairment	~50	Low ^†^	Similar responder rates as DS in real-world chart reviews up to 12 mo.; generalized tonic–clonic subtype shows higher responder rates (~56–63%).
Tuberous sclerosis complex (TSC)	Infantile onset, often with early developmental disruption and mTOR-driven cortical dysplasia	~50–60	Moderate ^‡^	LTE data (Expanded Access & real-world) show maintained efficacy across age, suggesting durable seizure suppression; subgroup analyses show similar efficacy regardless of age.
Developmental & other DEEs (broad DEE cohort)	Mixed (including LGS, DS, TSC, etc.)	~60	~21	Real-world study across DEEs shows higher overall responder rates, with ≥75% responders in ~21% after ~20 months; genetic etiologies correlated with better response
Adult focal epilepsies (non-DEE)	Typically, adolescence to adulthood onset; chronically remodeled, structurally consolidated circuits	25~40	~10	Long-term Expanded Access Program shows focal epilepsy responder rates around 25–40% with median follow-up of up to ~144 weeks; structural etiologies included
Non-syndrome drug-resistant epilepsy	Adult	~50	Low ^†^	Retrospective multicenter data show ~45–50% at 12 mo.; sustained reduction over time. Clobazam co-treatment did not significantly alter responder rates

Responder rate: % of patients with ≥50% seizure frequency reduction; ^†^ Low: under 10% complete seizure freedom in refractory syndromes; ^‡^ Moderate: occasional complete freedom in subsets, particularly TSC focal seizures.

**Table 2:** Developmental Stages, Observed Clinical Association, and Putative Biological Processes in Epilepsy Progression.

Developmental Stage	Epilepsy Syndrome Examples	Observed Clinical Association	Proposed Biological Process	Evidence Type
Neonatal/Early infancy	*SCN1A* (Dravet onset)	Fever-triggered clusters, rapid progression to DEE	Interneuron	RCTs/longitudinal
Early infancy	Dravet (*SCN1A*), West, *KCNQ2*	Earlier seizure control associated with improved cognitive outcomes	Activity-dependent network remodeling	Clinical observational
Early childhood/Adolescence	*PCDH19*, *WWOX*, *CDKL5*	Prolonged seizures linked to refractory epilepsy	Synaptic consolidation and E/I imbalance	Longitudinal cohorts
Later childhood/adolescence	*STXBP1*, Mixed DEEs	Persistent seizure burden, reduced plasticity, plateauing developmental gains	Network stabilization deficits and impaired cortical pruning	Clinical follow-up, Electrophysiological evidence

**Table 3:** Brain organoid developmental stages: in vivo equivalents.

Stage Proxy	Culture Days	In Vivo Equivalent	Key Neurodevelopmental Features	Epilepsy Relevance
Prenatal (mid-fetal) [70]	60–100	PCW 16–24	Neuroepithelial expansion, predominance early progenitors, limited neuronal maturation	Presymptomatic window of genetic epilepsies; early molecular pathogenesis modeling (e.g., SCN1A, KCNQ2)
Neonatal/perinatal [71,72,73]	120–200	Term newborn to 1 month	GABA switch initiation, initial network bursts, interneuron integration	Critical onset period for developmental and epileptic encephalopathies (e.g., KCNQ2-, WWOX-related DEE)
Early postnatal/Child-like [71,72]	250–350	~1–3 years	Mature synaptogenesis, theta-gamma oscillatory activity, astrocyte ramification	Modeling early childhood epilepsies, including Dravet syndrome and West syndrome; disorganized network dynamics
Adult-like [71,72]	400+	~4–10 years	Emergence of myelination-related features, stabilized oscillatory networks, reduced synaptic plasticity	Post–critical window chronic epilepsy phenotypes and irreversible circuit alterations

## Data Availability

No new data were created or analyzed in this study. Data sharing is not applicable to this article.

## Supplementary Materials

The following supporting information can be downloaded at: https://www.mdpi.com/article/10.3390/ijms27062899/s1.
