# Supplementary material for: Cannabidiol in Developmental Epilepsy: Organoid-Guided Precision Medicine Across Critical Neurodevelopmental Windows
Source: Int J Mol Sci. 2026 Mar 23;27(6):2899. doi: 10.3390/ijms27062899 (PMC13026608; doi:10.3390/ijms27062899)
Supplement: Supplementary file 1 [file ijms-27-02899-s001.zip › Table S1.pdf]

Table S1. Integrated molecular targets and multilevel mechanisms underlying the anticonvulsant effects of Cannabidiol

Molecular Target/ Pathway	Primary Action of CBD	Key Cellular & Synaptic Effects	Network-Level Consequences	Therapeutic Dimension	Developmental Relevance	References
CB1/CB2 receptors & FAAH	Negative allosteric modulation of CB1; FAAH inhibition increasing anandamide; CB2-mediated immune modulation	Reduced excitatory neurotransmitter release; enhanced endocannabinoid tone; suppression of microglial activation	Stabilization of synaptic transmission; attenuation of neuroinflammation	Acute seizure suppression + disease-modifying anti-inflammatory effects	Strong in pediatric developmental epilepsies; relevant in chronic adult epilepsy	[7,10]
GPR55 antagonism	Functional antagonism	Decreased Ca^{2+}-dependent glutamate release; reduced excitatory synaptic drive (↓ mEPSCs)	Dampening of hyperexcitable cortical and hippocampal circuits	Primarily acute anticonvulsant	Prominent in early-life hyperexcitable networks	[8,9]
TRPV1/TRPV2 channels	Transient activation followed by desensitization (TRPV1); modulation of Ca^{2+} homeostasis (TRPV2)	Net reduction in intracellular Ca^{2+} influx; neuroprotective signaling cascades	Suppression of repetitive firing and seizure initiation	Acute control with potential neuroprotection	High relevance during neurodevelopment when Ca^{2+} signaling is critical	[7,10]
ENT1–adenosine signaling axis	ENT1 inhibition elevating extracellular adenosine	Preferential A1 receptor activation; reduced presynaptic glutamate release; membrane stabilization	Shift toward inhibitory dominance in epileptic networks	Both acute suppression and homeostatic regulation	Effective across lifespan; especially protective in immature brain	[9,10]

5-HT1A receptors & PPARγ	Partial agonism and allosteric enhancement of 5-HT1A; transcriptional activation of PPARγ	Increased inhibitory neuromodulation; reduced oxidative stress and inflammatory gene expression	Improved network resilience and reduced seizure susceptibility	Primarily disease-modifying with supportive acute effects	Important for stress-sensitive pediatric epilepsies	[7,10]
