# Supplementary material for: Cannabidiol in Developmental Epilepsy: Organoid-Guided Precision Medicine Across Critical Neurodevelopmental Windows
Source: Int J Mol Sci. 2026 Mar 23;27(6):2899. doi: 10.3390/ijms27062899 (PMC13026608; doi:10.3390/ijms27062899)
Supplement: Supplementary file 1 [file ijms-27-02899-s001.zip › Table S2.pdf]

Table S2. Comparative mechanisms of action between conventional ASMs and CBD in epilepsy

Category	Conventional ASMs	Cannabidiol (CBD)
Overall pharmacological strategy	Primarily target-specific antiseizure suppression	Polypharmacological and network-modulating strategy
Main therapeutic goal	Direct suppression of ictogenesis and neuronal hyperexcitability	Modulation of epileptogenic processes in addition to seizure suppression
Principal molecular targets	Voltage-gated Na^+ channels, T-type Ca^{2+} channels, GABAergic signaling, SV2A	GPR55, TRPV1/TRPV2, ENT1-adenosine signaling, 5-HT1A, PPARγ, multiple ion channels
Mechanistic profile	Acts through relatively discrete and well-defined targets	Acts through multiple convergent pathways with limited target overlap with classical ASMs
Effects on synaptic/circuit function	Primarily reduces neuronal firing or enhances inhibition	Modulates excitation–inhibition balance, synaptic transmission, and pathological synchronization
Effects on Ca^{2+} homeostasis	Usually indirect or channel-specific depending on the drug class	Indirect stabilization of intracellular Ca^{2+} dynamics through TRPV modulation, GPR55 antagonism, and network effects
Effects on neuroinflammation	Generally limited or secondary	Includes anti-inflammatory actions through modulation of microglial activation and cytokine signaling
Effects on oxidative stress/mitochondria	Not a major shared therapeutic feature across most ASMs	Potential antioxidant and mitochondrial protective effects
Clinical positioning	Established first-line or adjunctive therapies	Currently approved mainly as adjunctive therapy in selected developmental epilepsies
Relevance in drug-resistant epilepsy	May be insufficient when epileptogenesis extends beyond classical ion-channel dysfunction	May provide complementary benefit in drug-resistant epilepsy because of broader mechanistic coverage

This table summarizes the principal mechanistic differences between conventional ASMs and CBD based on the mechanisms and experimental evidence discussed in Sections 3 and 4

ASM, Antiseizure medication; CBD, Cannabidiol; GABA, Gamma-Aminobutyric Acid; SV2A, Synaptic vesicle protein 2A; GPR, G protein-coupled receptor;
TRPV, Transient receptor potential vanilloid channels; ENT1, Equilibrative nucleoside transporter-1; HT1A, Hydroxytryptamine; PPARγ,, Peroxisome proliferator-activated receptor-γ
